# Recent Advances in Cancer Therapy Based on Dual Mode Gold Nanoparticles

**DOI:** 10.3390/cancers9120173

**Published:** 2017-12-19

**Authors:** Ellas Spyratou, Mersini Makropoulou, Efstathios P. Efstathopoulos, Alexandros G. Georgakilas, Lembit Sihver

**Affiliations:** 1 2nd Department of Radiology, Medical School, National and Kapodistrian University of Athens, 12462 Athens, Greece; spyratouellas@gmail.com (E.S.); stathise@med.uoa.gr (E.P.E.); 2 Department of Physics, School of Applied Mathematical and Physical Sciences, National Technical University of Athens, 15780 Athens, Greece; mmakro@central.ntua.gr (M.M.); alexg@mail.ntua.gr (A.G.G.); 3 Atominstitut, Technische Universität Wien, Stadionallee 2, 1020 Vienna, Austria

**Keywords:** gold nanoparticles, ionizing radiation, photothermal therapy, radiotherapy, radiosensitizing

## Abstract

Many tumor-targeted strategies have been used worldwide to limit the side effects and improve the effectiveness of therapies, such as chemotherapy, radiotherapy (RT), etc. Biophotonic therapy modalities comprise very promising alternative techniques for cancer treatment with minimal invasiveness and side-effects. These modalities use light e.g., laser irradiation in an extracorporeal or intravenous mode to activate photosensitizer agents with selectivity in the target tissue. Photothermal therapy (PTT) is a minimally invasive technique for cancer treatment which uses laser-activated photoabsorbers to convert photon energy into heat sufficient to induce cells destruction via apoptosis, necroptosis and/or necrosis. During the last decade, PTT has attracted an increased interest since the therapy can be combined with customized functionalized nanoparticles (NPs). Recent advances in nanotechnology have given rise to generation of various types of NPs, like gold NPs (AuNPs), designed to act both as radiosensitizers and photothermal sensitizing agents due to their unique optical and electrical properties i.e., functioning in dual mode. Functionalized AuNPS can be employed in combination with non-ionizing and ionizing radiation to significantly improve the efficacy of cancer treatment while at the same time sparing normal tissues. Here, we first provide an overview of the use of NPs for cancer therapy. Then we review many recent advances on the use of gold NPs in PTT, RT and PTT/RT based on different types of AuNPs, irradiation conditions and protocols. We refer to the interaction mechanisms of AuNPs with cancer cells via the effects of non-ionizing and ionizing radiations and we provide recent existing experimental data as a baseline for the design of optimized protocols in PTT, RT and PTT/RT combined treatment.

## 1. Introduction

With the rapid development of nanotechnology, there has been a revolutionary growth in proposals of smart nanosystems for use in cancer imaging, targeted administration of drugs and post-therapeutical monitoring of cancer regression in oncology. For example, nanoparticles (NPs), such as gold, gadolinium, hafnium, silicon, ferromagnetic and polymer NPs, quantum dots, nanorods, nanotubes, liposomes and dendrimers have been examined and utilized—at least preclinically—as drug delivery systems in personalized nanomedicine. Typically, NPs, with their dimensions of less than 100 nm, exploit the increased vasculature permeability and decreased lymphatic function of tumors. One “competitive edge” of NPs is that they can be attached to specific cancer cell targets with non-invasive implementation, increasing the cellular uptake efficiency, selectivity and localization in tumor cells and tissues. Metallic NPs, semiconductor quantum dots and carbon nanotubes have been used as light-activated heating nanosystems which can be incorporated into tumors allowing high heat administration in the tumor area in hyperthermia treatment, and minimizing the damage in the surrounding healthy tissue [1,2]. However, one of the main challenges to this approach is the “thermotolerance” of the tumors whereby cells or tissues become resistant to elevated temperatures as a result of prior or continuous exposure to hyperthermia. Metallic NPs can also act as radiosensitizers in radiotherapy (RT) treatment providing “dose enhancement”. One challenge in RT is how to deal with the radiation resistance of hypoxic tumor cells. This is especially a problem when using conventional low linear energy transfer (LET) X-rays/γ-rays, and it is not possible to increase the radiation dose to levels high enough to kill the hypoxic tumor cells while sparing normal proximal tissues. The use of radiosensitizers could be a way to enhance the radiotherapy efficacy and make it more specific against the malignant tumor cells while drastically reducing the damage to surrounding normal tissues. Among the various types of NPs, gold NPs AuNPs (or GNPs) have been brought to the forefront of cancer research due to their unique properties, exhibiting a combination of characteristic physical, chemical, optical and electronic properties.

### The Basic Physics of Interactions of AuNPs with Ionizing Radiation and Laser Radiation

As the prospective for nanotechnology in personalized medicine is undergoing considerable progress, several studies have aimed at investigations of the actions of both ionizing and non-ionizing radiation on nanosystems, regarding the elucidation of the principal mechanisms of radiation—NP interaction, depending on both the physico-chemical properties of the NPs and the irradiation parameters. 

The main physical mechanisms through which ionizing radiation interacts with NPs (in the keV range) are the Compton and photoelectric phenomena, where an incident photon can either be partially or fully absorbed by an atom, resulting in the ejection of an electron. The parameters and the relevant mechanisms of NP and X-ray interactions are the subject of numerous reports and the interested reader should refer to recent reviews of [3,4,5]. Having, as a starting point, the basic physical interactions of X-rays with biological tissues (the photoelectric and Compton effects), it is expected that exposure of high atomic number (Z) NPs to X-rays enhances the photoelectric and Compton effects, when compared to exposure of relatively light elements, such as hydrogen, carbon, and oxygen, in biological tissues. This would lead to an increased radiation therapy efficiency. However, the increase in the interaction cross-sections of AuNPs vs. soft tissue decreases with increasing X-ray energy, and it has indeed been found that the energy of the radiation plays a major role in the radiosensitization effect [3].

Obviously, the energy of the X-rays is important, since the photoelectric effect is dominant at lower energies and prevails until the photon energy reaches a medium energy (e.g., around 500 keV for Au) with a cross section varying with Z^4^ or Z^5^, depending on the target material, and is enhanced by an increased absorption by electron shells (K, L, M, etc.) at low energies [4]. When using X-rays, mainly the inner electron shells are ionized and this creates cascades of both low and high energy Auger electrons. Increasing the atomic number Z of the NP is enhancing the photoelectric and Compton effects when they are exposed to X-rays. That is why high Z NPs are more radiosensitizing when using X-rays than low-Z NPs. Therefore, an approach to maximize the differential response between the tumor and normal tissue, termed therapeutic ratio, is through the introduction of high-Z material into the target. AuNPs with Z = 79 are promising radiosensitizers in this regard due to their high atomic number and mass energy coefficient relative to soft tissue [6]. Haume et al. [3] have presented a comprehensive overview on the physico-chemical properties of NPs (shape, size, surface charge and coating, concentration and NP toxicity) and their role in radiosensitization. The concentration and the physicochemical characteristics of the NPs, as well as their location inside the cell [5,7] influence the radiosensitizing action of NPs.

The interaction of non-ionizing light photons with small particles depends strongly on the dimension, shape, surface treatment and composition of the particles, as well as on the composition of the medium in which the particles are embedded. If we consider the special case of metal NPs, absorption, scattering and extinction mechanisms of laser radiation by a single metal nanoparticle determine the subsequent photophysical and photochemical processes [8]. Solving the problem of absorption and scattering of electromagnetic radiation by a small particle involves solving Maxwell’s equations with the correct boundary conditions. To solve Maxwell’s equations, various analytical or numerical methods can be used [9]. The most popular and simple analytical method is the one developed in 1908 by Gustav Mie [10], while a complete derivation of Mie’s theory is given by Bohren and Huffman [11]. Both these works have even been cited in recent years by [12,13]. The general Mie theory of light scattering by a spherical metal particle states that the scattering cross-section of a nanoparticle of radius R, much smaller than the wavelength of the incident light λ (R << λ), varies as R^6^, while its absorption cross-section varies as R^3^ [12]. This implies that absorption is more important than scattering for very small particles.

When a metallic nanoparticle interacts with light (an electromagnetic wave), the oscillating electric field causes the conduction electrons to oscillate coherently. The optical properties of metal NPs, are therefore, as a consequence of their reduced dimensions, dominated by the coherent collective oscillation of their conduction band electrons. Collective excitations of conductive electrons in metals are called “plasmons” [9]. Depending on the boundary conditions, it is commonly accepted to distinguish between bulk plasmons (3D plasma), surface propagating plasmons or surface plasmon polaritons (2D films), and surface localized plasmons (NPs) [14]. Surface plasmon resonance (SPR) is a nanoscale electronic effect that causes metallic NPs to absorb and scatter electromagnetic radiation of wavelengths considerably larger than the particles themselves [15]. Metallic NPs can resonate plasmonically under exposure to light when the laser wavelength corresponds to their SPR. The resonated NPs convert the electromagnetic energy into heat via a non-irradiative process causing hyperthermic damage to cancer cells. The plasmonic oscillations can be tuned in a wide spectral range from visible to near infrared (NIR) when the NPs shape deviates from the highly symmetric nanospherical shape to nanoshells (AuNSs), nanocages (AuNCs) and nanorods (AuNRs) [2]. For example, the SPR band shifts to the NIR region by changing the shell to core ratio, the aspect ratio and the particle size, wall thickness and wall porosity for AuNSs, AuNRs and AuNCs, respectively [2]. The optical properties and efficiencies of the gold NPs depend strongly on their sizes, geometries, surface modifications and the refractive index of the surrounding medium [16] together with laser irradiation parameters such as laser wavelength, pulse duration, irradiation and time.

Recently, a fast developing research field is to combine laser based photothermal therapy with conventional ionizing radiation therapy using NPs. As we mentioned above, besides their photothermal properties, NPs can also act as radiosensitizers in RT providing “dose enhancement”, increased cellular uptake efficiency, selectivity and localization in tumor cells and tissues. When internalized into tumor, these NPs increase the cell killing after irradiation due to its properties, including high atomic number (Z), high absorption coefficient and good biocompatibility. The NPs are straightforward to synthesize, in a wide range of sizes, and their surface functionalization can easily be modified by the attachment of ligands required to target cancer cells, and organelles therein, or increase the life time in the bloodstream [3,17]. Thus, lower radiation doses can be used to eradicate tumors with the same efficiency, or even better, while decreasing the damage to surrounding normal tissues.

## 2. Application of Interaction of AuNPs with Non-Ionizing Radiation (Non-IR) in Cancer Therapy

### 2.1. Introductory Remarks about Non-IR Cancer Treatment Modalities 

Human beings, and other mammals, have a recorded history of cancer incidence throughout history. Cancer is treated with both invasive and non-invasive treatment modalities, such as surgery, radiation therapy, chemotherapy, as well as other therapeutic modalities (e.g., immunotherapy, hormone therapy, biological therapy, thermotherapy, photodynamic therapy), and a combination of these (e.g., radiosurgery). Undoubtedly, in the anti-cancer fighting armamentarium, radiation therapy (RT) is widely used for curative treatment. Ionizing radiation mainly kills the cells either by apoptosis or mitotic cell death (mitotic catastrophe). Following radiation, necrosis can also be detected to some extent in both cancer cell lines and normal tissues, but normally less frequently than apoptosis and mitotic cell death. Mitotic cell death occurs during or after aberrant mitosis (cell division) and is caused by mis-segregation of chromosomes leading to formation of giant cells with aberrant nuclear morphology and multiple nuclei. Normally cell death through apoptosis is considered most preferable death since apoptosis is characterized by cell shrinkage and formation of apoptotic bodies. Necrosis is less orderly than apoptosis, and in contrast with apoptosis, cleanup of cell debris by phagocytes of the immune system is generally more difficult, as the disorderly death generally does not send cell signals which tell nearby phagocytes to engulf the dying cell. However, cell death through necrosis produces an inflammatory response which might be beneficial for the treatment. However, RT based on high-energy ionizing radiation (X-rays, gamma rays, electrons, or ions) is not the only option, as nowadays there are possibilities to develop novel cancer treatment strategies specifically based on novel biophotonic principles, e.g., on the use of non-ionizing photon RT [18]. In addition, the revolution in the fields of physics and technology of monochromatic and coherent laser light, has open up new opportunities in oncology, e.g., for diagnosis, drug delivery and therapy. The possibility to combine different monitoring modalities [19] with therapy in the field of *theranostics*, which is the association between thera(peutics) and diag(nostics) [20,21] has also opened up new opportunities for the treatment of cancer. This interdisciplinary approach is a very promising challenge for both the researchers and the physicians and, with the rapid development of nanomedicine and pharmaceutical technology in the last decades, a revolutionary growth of theranostics methods have been proposed using several different nanosystems which have multiple functions: early cancer detection, targeted drug delivery, and monitoring of the cancer progression [22]. Among the new radiation therapy approaches, PhotoDynamic Therapy (PDT) is a non-invasive or minimally invasive biophotonic modality, based on the simultaneous action of the following three factors: (i) photosensitizing agents, able to photochemically eradicate malignant cells; (ii) generation of highly reactive singlet oxygen; and (iii) non-thermal monochromatic light irradiation. Briefly, PDT’s activity is based on the photo-generation of singlet oxygen ^1^O_2_ or reactive oxygen species (ROS), through the photosensitizer excitation due to photons absorption, which can cause cellular destruction [23]. PTT is an extension of PDT which does not require oxygen, as the stimulated photosensitizing agent release vibrational energy (heat) which kills the targeted cells. It is worthy to mention that ancient Greeks, Romans, and Egyptians used heat to treat breast masses, and this is still a recommended self-care treatment for breast engorgement. 

### 2.2. Photothermal Therapy (PTT) and Gold NPs: Advantages and Limitations

PTT is a newly developed and encouraging type of hyperthermia which employs photon energy of laser activated photoabsorbers to generate heat sufficient to cause cells’ destruction. The hyperthermia treatment can also be conducted with radiofrequency, microwave, ultrasound and laser which are less invasive treatments. Hyperthermia is connected to cell death with three mechanisms, cell apoptosis and cell necrosis and relatively recently with necroptosis which is a type of programmed necrosis [24]. Cell apoptosis takes place when the heating temperatures range from 41 °C to 47 °C. Excessive cell necrosis is occurred by heat shock for temperatures usually higher than 50 °C, producing a much quicker cell death than apoptosis and is based on protein denaturing [25]. Hyperthermia is a treatment that can minimize the toxicity of chemical and radioactive agents, destroying diseased cells, without harming the healthy ones, but still requires the invasion with a probe to heat the malignant cells. The recent development of AuNPs, as probes capable of efficient heat generation under laser irradiation renders PTT is a very promising tool. Metallic NPs, semiconductor quantum dots, polymeric NPs, nanographene sheets and, carbon nanotubes have been used as light-activated heating nanosystems which can be incorporated into tumors allowing high heat deposition in the tumor area at low laser intensities and minimizing the damage in the surrounding healthy tissue [1,2,26].

Among the various types of NPs, AuNPs have been brought to the forefront of cancer research mainly due to their strongly enhanced and tunable optical properties. AuNPs can accumulate in the tumor tissues via the passive mechanism referred as “Enhanced Permeability and Retention, (EPR) effect” [27] or by active targeting by conjugation of a chemical moiety that is over expressed in cancer cells (monoclonal antibody, folic acid for cancer treatment). In order for the NPs to find and accumulate at their target cells, through the systemic route, they need to overcome several extra- and intracellular barriers by fulfilling several major prerequisites including: (a) their outer periphery must be inert in order to avoid the activation of the reticuloendothelial system (RES) so that they can circulate in the blood compartment for prolonged periods of time increasing the probability of reaching the pathological cells; (b) their dimensions should be close to 100 nm in order to escape the blood compartment and locate the pathological cells; (c) after internalization in the pathological cells, the NPs should be to overcome multiple obstacles related to: (i) cell surface binding; (ii) cellular uptake; (iii) escape from lysosomes/endosomes; and (iv) association with a particular subcellular location, such as nuclei or mitochondria [28].

The sizes of the AuNPs are critical: as the diameter of the AuNPs e.g., nanoshells, become smaller, the plasmon resonance wavelengths are significantly less optically tunable than those of larger diameter nanoshells. Furthermore, NPs smaller than ~10 nm are easily cleared from circulation via the kidneys and thus do not have adequate residence time to accumulate in target tissue. The concentration, the toxicity, the degree of aggregation, the circulation time and the subcellular location of the NPs affect PTT efficacy. Some NPs are not toxic at low concentrations but when the incubation time or concentration is increased they show a degree of toxicity. In addition, high NP concentrations increase the probability of aggregation, which is a critical point since the optical properties of the NPs are strongly deteriorated by the presence of aggregation [2]. The surface treatment of the NP is therefore of great importance. Many studies indicate that AuNPs reveal a more effective PTT when they are located in the cell membrane than when they are located in the cells, while some other studies have focused on the potential benefits of targeting AuNPs to specific cell parts, e.g., the nucleus and the DNA [28]. When photothermal treatment is conducted by continuous-wave (CW) laser irradiation, AuNPs usually localized in the cytoplasm are more effective in inducing cell destruction than AuNPs localized at the nucleus [29]. On the other hand, high localization of AuNPs at the nucleus is needed when nanosecond pulsed laser is used, suggesting that nonlinear absorption processes enhance the plasmonic field. CW laser induces cell death via apoptosis, while the nanosecond pulsed laser leads to cell necrosis [29]. These findings indicate that the cell death mechanism depends of the different mechanism of laser-cells interactions. A number of research projects have been carried out using different types of AuNP: nanospheres, nanoshells, nanocages and nanorods to overcome the limitations for targeted cancer therapy by photothermal treatment [30,31]. 

#### 2.2.1. Photothermal Therapy and Gold Nanoshells

Plasmonic photothermal therapy using gold-based nanosphere contrast agents was reported for the first time by Lin and co-workers in 2003 [32] in combination with a nanosecond visible pulsed laser. T lymphocytes were labeled with anti-human CD8 mouse IgG and then incubated with 30 nm gold particles conjugated to anti-mouse IgG. The cells were irradiated with 100 laser pulses (565 nm wavelength, 20 ns pulse duration) at an energy fluence of 0.5 J/cm^2^ leading to the destruction of more than 90% of the cells according to cell viability tests. Photothermal destruction has also been performed successfully on squamous carcinoma cells lines (HSC 313 and HOC 3 Clone 8) and a benign epithelial cell line (HaCaT) after incubation with ~40 nm anti-EGFR antibody conjugated Au nanospheres [33] and CW laser irradiation at 514 nm, for 4 min at power densities of 57 W/cm^2^ and 25 W/cm^2^, respectively. No cell death was observed in the absence of NPs at four times the energy required to kill the malignant cells with bonded anti-EGFR/Au conjugates.

Plasmonic hollow gold nanoshells coated with PEGylated thermosensitive lipids were used as an efficient vehicle to co-deliver two drugs, bortezomib and gemcitabine (GNS-L/GB), for combinational chemotherapy and photothermal therapy of pancreatic cancer. GNS-L/GB showed synergistic cytotoxicity and improved internalization by MIA PaCa-2 and PANC-1 cells. High-powered near-infrared continuous wave laser (λ = 808 nm) at 4 W/cm^2^ effectively killed cancer cells via the photothermal effect of GNS-L/GB, irrespective of cell type in a power density, time-, and GNS dose-dependent manner. These results suggest that this method can provide a novel approach to achieve synergistic combinational chemotherapy and photothermal therapy, even with resistant pancreatic cancer [34].

For in vivo applications, PTT using NIR light is favorable as it penetrates deeper into the tissue due to minimal absorption by the major components of water and hemoglobin. Hirsch and coworkers [35] were first to develop NIR PTT using PEG-coated gold nanoshells (AuNSs) for human breast cancer cells morbidity in vitro after exposure to CW NIR light (at λ = 820 nm) at a power density of 35 W/cm^2^ for 7 min. In vivo studies in mice show that by injecting the PEG-AuNSs interstitially into the tumor volume, and then irradiating with NIR light (at λ = 820 nm) at 4 W/cm^2^, temperatures capable of inducing irreversible tissue damage (ΔT = 37.4 ± 6.6°C) within 4–6 min were raised. However, the desired biodistribution of AuNSs and the prolonged blood circulation remains a challenge. Recently, macrophage cell membrane (MPCM)-camouflaged gold nanoshells served as new generation agents for in vivo photothermal cancer therapy. The tumor area was irradiated by an 808 nm NIR laser at 1 W/cm^2^ for 5 min after 20 min of intravenous injection of the MPCM-AuNSs in mice and subsequent tumor volume and body weight measurements were performed every day until the 25th day. This surface functionalization provided active targeting ability by recognizing tumor endothelium and thus improved tumoritropic accumulation, enhanced in vivo blood circulation time and local accumulation at the tumor when administered systematically and improved the efficacy of PTT [36]. Tumor necrosis factor-alpha coated gold nanospheres (Au-TNF) heated by nanosecond laser pulses demonstrate higher therapeutic efficiency against murine carcinoma [37]. Au-TNF are already in phase I trials in humans as nanodrugs emphasizing the clinical relevance of PT nanodrug.

Recently, hollow gold nanoshells with cores densely packed with small interfering RNAs have been efficiently used in photothermal treatment in a nude mice model. The thermal resistance of cancer cells is closed related with the over expression of the heat shock proteins 70 (HSP70s), which are abnormally upregulated when cells are under lethal stresses. Under NIR light irradiation, efficient down regulation of Hsp70 is achieved since the small interfering RNAs can detach form the gold surface, escape from endosomes and target the mRNA leading to down-regulation of HSP70 gene expression. Meanwhile, the temperature increases for hyperthermia therapy due to the high photothermal efficiency of the nanoshells [38].

#### 2.2.2. Photothermal Therapy and Gold Nanorods

NIR PTT based on gold nanorods (AuNRs) was firstly demonstrated using in vitro studies where AuNRs functionalized with anti-EGFR antibodies were bound to human oral cancer cells [31]. After exposure to NIR light (λ = 800 nm) at 10 W/cm^2^ the malignant cells were clearly injured while the threshold for normal cells was found at 15 W/cm^2^ [33]. Since then, several in vitro and in vivo studies indicate the potential of AuNRs due to their unique properties [39,40]. AuNRs absorb light in the NIR region that penetrates deeper in tissues with higher spatial precision. The absorption range can be also fine-tuned by adjusting the aspect ratios of AuNRs.

The feasibility of the plasmonic PTT as applied to natural tumors in the mammary glands of dogs and cats were demonstrated after injection of pegylated-AuNPS and laser irradiation after 5 min using an 808 nm diode laser with a power density 5.8 W/cm^2^. The results of PTT in animals, histopathology evaluation, blood analysis, and X-ray diffraction show a complete regression in the cancer grade after the third treatment with no obvious changes in liver and kidney functions and no metastasis after 1 year of the treatment [41]. Monosaccharide-imprinted gold nanorods using sialic acid (SA) induced a significant decrease of a tumor examined two weeks after photothermal treatment carried out on HepG-2 tumor-bearing mice [42]. Fluorescence images of a tumor-bearing mouse at 24 h post injection of fluorescence-dye doped SA-imprinted AuNRs show a good targeting selectivity to cancer cells. According to the anti-tumor tests, SA-imprinted AuNRs exhibited good biocompatibility and also high photothermal effect. In vitro photothermal test explored with HepG-2 cells and L-02 cells incubated with SA-imprinted AuNpS show that after irradiation with a 750 nm laser beam for different durations (2, 4, 6, 8 min) the most HepG-2 cells were found dead while the viability of L-02 cells was still higher at 85% as it was given by MTT assay.

Anti-EGFR-conjugated AuNRs (antiEGFR-GN) upon NIR-PTT exerted synergetic anti-proliferative and apoptotic actions through upregulation of HS70 protein and cleaved caspase-3, downregulation of Ki-67 and EGFR, and inhibition of several intracellular signaling molecules (mTOR, AKT, ERK1/2 and FAK) (Table 1) [43]. AuNRs, coated with dipicolyl amine (DPA), which forms stable complexes with Zn^2+^ cations, results to Zn(II)/DPA-GNRNPs which recognize phosphate-containing molecules including siRNA. Zn(II)/DPA-GNR presents strong complexation with the anti-polo-like kinase 1 siRNA (siPLK), which is used for gene silencing. The SiPLK/Zn(II)DPA-GNRs nano-complex was efficiently delivered siRNA into the cancer cells in vitro and in vivo, in PC-3 tumour mouse model (Table 1) [44].

## 3. Application of Interaction of AuNPs in Combination with Ionizing Radiation (IR) in Cancer Therapy

### 3.1. The Combination of Metallic NPs with Radiotherapy

Metallic NPs can combine multiple functionalities to target and treat cancer cells. Besides their phototothermal properties, they can also act as radiosensitizers for RT due to their unique electrical properties. IR therapy is currently a major and most applicable modality for cancer treatment following surgery employed in the treatments of more than 50% of all treated cancer patients [55]. However, it is still a great challenge to confine the curative dose of radiation within the tumor tissue while sparing the adjacent normal tissues. An important disadvantage of photon therapy is that cancer tissues can have, or develop, resistance to radiation. Especially hypoxic tumor cells show radiation resistance to photon treatment. This is because in the hypoxic cells there is a decreased production of reactive oxygen species (ROS) in response to irradiation, which leads to decreased levels of oxidative stress and therefore a decreased level of apoptosis compared with irradiated normoxic cells. Moreover, due to the physical extent of this tissue, it can be difficult to effectively irradiate the whole tumor, which may lead to its regeneration. To make predictions of radiation effects, popular simulation models of the effect of RT, microdosimetric kinetic model (MKM) originally developed by Hawkins et al. [56,57] and later modified by different groups [58], has been used also in the case of AuNPs in conjunction with the local effect model (LEM) [59,60,61]. The MKM model gives the dependence of the relative biological effectiveness (RBE) in the limit of zero doses on the linear energy transfer (LET) while the foundation of the LEM is that the local biological response to IR is expected to be equal for equal doses and independent of the type of radiation.

Based on the fact that AuNPs, have large interaction cross sections with X-ray radiation, at least up to about 1 MeV, the dose delivery in the area of gold NPs sometimes increases dramatically [3]. This dose enhancement facilitates a possible strategy to address the issue of radio-toxicity and adverse effects. The use of radiosensitizers in the tumor that confer additive and synergistic advantages to the tumor-killing effect of IR, significantly affects in various cases of tumor treatment [62]. If one can achieve radiation dose enhancement, lower radiation doses can be used to eradicate tumors with the same efficiency, or even better, while decreasing the damage to surrounding normal tissues [63]. A number of reviews have been published to date supporting the use of gold NPs in targeted cancer therapy and the augmentation of the effect of radiation (both IR and NIR) [3,64,65,66]. 

During the last decade, several in vivo and in vitro preclinical studies have been conducted regarding the applications of metal NPs in radiation therapy [67,68]. McMahon [59] and co-workers irradiated the breast cancer cell line MDA-MB-231 with 6 and 15 MV X-rays produced by a linear accelerator (LINAC) with and without the addition of 1.9 nm AuNPs. At MV energies, a significant radiosensitization is not expected based on the ratio of mass energy absorption coefficients of gold. However, the results showed that the sensitization enhancement ratio (SER) was 1.24 ± 0.05 at 6 MV and 1.18 ± 0.04 at 15 MV. Therefore, the contrary to the initial thought that AuNPs could be used only for physical “dose enhancement”, by exploiting the enhanced photoelectric absorption of gold, it seems that both chemical and biological mechanisms contribute to their radiosensitization. 

Gold nanorods modified with argine (R)-glycine (G)-aspartate (D) (RGD) peptide demonstrate enhanced radiotherapy of melanoma in cancer cells. The results show an enhancement in the radiosenstitivity of A375 cells with a dose modifying factor of 1.35 at 6 MV X-ray and at dose 4 Gy. RGB is the most effective peptide presented in many extracellular matrix proteins and can interact with integrin receptors α_v_β_3_ at focal adhesion points. Integrin α_v_β_3_ expression affects tumor radiosensitivity and it is also associated with acquired radioresistance through the upregulation of integrin expression by radiation [69]. Griffin and his group have recently showed that tumor necrosis factor-alpha coated gold nanospheres (Au-TNFα) combined with single or fractionated high-dose radiation therapy reduce effectively the tumor interstitial fluid pressure (IFT) and tumor growth in 4T1 murine breast tumour model, giving hopes for clinical translation [70].

The shape/geometry of the AuNPs is found to be critical in RT, as AuNPs coated with the same PEG molecules and a similar average size (~50 nm) exhibit different cellular uptake and sensitization enhancement ratios in KB cells. The SERs were calculated 1.62, 1.37, and 1.21 corresponding to the treatments upon X-ray irradiation (6 MV) at dose of 4 Gy of gold nanospheres, gold nanospikes and gold nanorods, respectively. The results indicated that gold nanospheres showed a higher anticancer efficiency than both AuNSs and AuNRs [71].

The contribution of NPs to radiosensitization has been demonstrated for a range of different external beam radiation types, including kilovoltage (kV) and megavoltage (MV) photons, MeV electrons and heavy charged particles [53,72,73], suggesting broad in vivo applications. The last mentioned RT approach is the ion-based RT consisting of ions of hydrogen (protons), helium, carbon, or oxygen. In radiation therapy, protons and helium ions are often referred to as “light ions” and the others as “heavy ions” [3]. Radiation therapy with protons and carbon ions is usually also called by the collective names “particle therapy” or “hadron therapy”. Protons and light ions have unique physical properties, as they penetrate to the patient body with minimal lateral diffusion, depositing most of their energy at the end of their range (in the so-called Bragg peak), protecting in this way the surrounding healthy tissues. Therefore, particle therapy is particularly useful in pediatric radiation oncology.

For irradiation of biological samples, and especially for the applicability of these experiments to patient proton-based therapy, the positioning of the biological samples is very important. The most usual setup and of clinical relevance is the utilization of three points: entry (or plateau), center of spread-out Bragg peak (SOBP) and towards the end of the track (distal end) [74,75]. Logically, the maximization of LET around the Bragg peak region is expected to induce lesions of high complexity. Indeed and based on DSB measurement using surrogate markers like 53BP1, a high persistence of damage is shown when cells are exposed in this SOBP region compared to the entry and a significant increase in the RBE for cell survival reaching values of about 2 [74,75]. In accordance to these experimental studies, recent work by Nikitaki et al. [76], simulating the complex DNA damage induction along the central axis of a representative 200.6 MeV pencil beam (2 mm spot size, 5 mm FWHM) as calculated using Monte Carlo DNA damage simulation software (MCDS) and based on the actual beam energy-to-depth curves, a variation of clustered DNA damage (DSBs and non-DSB clusters) is predicted. A local maximum of clustered DNA lesions around the SOBP region, with maximum complexity, is found which underlies the importance of the SOBP region high-LET. Monte Carlo simulations used to assess the dose enhancement of AUNPs for proton therapy showed that proton therapy can be enhanced significantly only if the GNPs are in close proximity to the biological target [77,78]. According to Schuemann et al., the dominant process for proton interactions with gold is the production of secondary electrons via small angle scattering due to the higher density of gold as compared to soft tissue (19.3 g/cm^3^ vs. ~1 g/cm^3^) [79]. 

The dose enhancement caused by AuNPs in radiation therapy differs for photon and proton radiation. The enhancement effect has been simulated [78] for protons, kVp photons and MV photons, and the simulations conclude that AuNPs have the potential to enhance radiation therapy depending on the type of radiation source. In some cases, kilovoltage energies result in higher dose enhancement factors than megavolt energies [6]. For the same amount of energy absorbed in the AuNP, interactions with protons, kVp photons and MV photons produce similar doses within several nanometers of the AuNP surface, and differences are below 15% for the first 10 nm. However, secondary electrons produced by kilovoltage photons have longer ranges in water as compared to protons and MV photons, e.g., they cause a dose enhancement 20 times higher than the one caused by protons 10 μm away from the AuNP surface [78].

AuNPs’ surface, depending on the subcellular localization, can trigger radiochemical sensitization of DNA [80], catalysis [81] and radical generation [82,83] upon IR exposure. Electrons with energies below the ionization threshold (e.g., 12 eV) levels fail to produce considerable secondary electrons through interaction with AuNPs, but they can cause a great deal of DNA damage [80]. For example, and as mentioned above, a DNA damage enhancement was showed using HeLa cancer cells, 50 nm AuNPs and a clinical 6 MV beam for various delivery parameters and depths [73]. In this case, increased γH2AX staining was detected suggesting an increased induction of DSBs. In addition, the role of AuNP charge seems to play a significant role in the overall outcome. 

The chemical radiosensitization changes depend on the size and charge of AuNPs [84]. In vitro studies have showed that cell survival decreases with increased cellular internalization of AuNPs and the closer AuNPS are located to the nucleus of target cells [63,85]. Recently, simulations with a model considering the dosimetric characteristics of AuNPs, and the Local Effect Model (LEM) [59], showed very good agreements with experimental survival curves for x-rays assuming in all cases a perinuclear distribution of AuNPs.

In order to further understand the mechanism of AuNP dose enhancement using proton radiation, the group of Lin et al. [78] has recently investigated (using a modified LEM based on Monte Carlo simulations) several potential configurations for AuNP-induced radiosensitization for three particle sources: brachytherapy or superficial RT, AuNPs as tumor vascular-disrupting agents, RT application with in situ dose-painting using AuNPs and customizable RT enhancement of wet age-related macular degeneration using AuNPs. Lin et al. consider these AuNPs plus RT modalities as promising emerging procedures, representing three clinically relevant scenarios [86]. The impact of the beam quality on megavoltage RT treatment techniques, in combination with AuNPs for dose enhancement, as a function of irradiation conditions and potential biological targets was reported recently by Tsiamas et al. [87]. Moreover, several theoretical and experimental and/or preclinical studies are concerned with the interdisciplinary area of “gold and other metallic NPs” in combination with “RT using protons and other ions”. The interested reader could study a series of comprehensive relative reviews [3,6,79,88]. Indeed, a PubMed search for scientific information and pre-clinical/clinical applications in this interdisciplinary area reveals an ongoing increased number of publications, reflecting the great interest of the biophysical and medical community in this area.

Definitely, NP assisted radiation therapy opens up new perspectives in the fight against cancer. However, there are some problems and limitations that prevent it from moving from bench to bedside. The mechanism of nanoparticle-mediated radiosensitization seems to be simple, since the main target for ionizing radiation is the nuclear DNA and NPs escalate the attack of radiation on this molecule. However, the biological mechanisms of nanoparticle-mediated radiosensitization remain more obscure [89] since many reports failed to demonstrate augmented nanoparticle-mediated DSB damage though a significant radiosensitizing effect had occurred [89]. 

Among the most important things to be mentioned is the need for ensuring the biocompatibility and the effective delivery of NPs to the target in the patient’s body. It was proposed that the enhancement of the uptake of NPs by cancer cells can be reached via passive targeting, active targeting, or a combination of both strategies and functionalization through coating with biocompatible polymer and conjugation with tumor targeting agents [90]. Some more recent studies turn to other nanostructures called hydrogenated nanodiamonds (H-NDs) which has been proposed as radiosensitizers, as they exhibit excellent biocompatibility, negative electron affinity that confers a high reactivity with oxygen species (ROS) and an increased induction of DNA DSBs. The characteristics of H-NDs allows electron emission from H-NDs following irradiation by photons and in consequence may enhance the effects of radiation on cancer cells [91,92].

This year, in an attempt to identify if physical properties are correlated with the survival fraction of cells exposed to low-energy protons in combination with metallic NPs, an interdisciplinary group in Belgium [92] performed Monte Carlo simulations (based on the latest version of the Geant4 toolkit), assessing also in their study the influence of nanoparticle coating on dose enhancement. They conclude that, although significant efforts will be required to unravel the relevant mechanisms of action, ROS-induced damage and local heating generated from metallic NPs are likely to become fields of importance in radiation therapy research, despite a DNA-centered dogma. Another limitation for nanoparticle’s assisted hadron (proton, carbon) RT is related to the cost and huge size of a proton-based cancer therapy facility. However, there are several hard works to “shrink” this problem, by the ongoing research and development efforts on the so called “laser-driven ion beam radiation therapy”. According to Ledingham et al. [93], the overall challenge for laser-driven ion beam RT is “*to develop well-controlled, reliable energetic ion beams of very high quality that can meet stringent medical requirements with respect to physical parameters and performance*”, representing a viable alternative in the 21th century’ RT.

### 3.2. The Combination of Photothermal Therapy with Radiotherapy

The combination of PTT with RT can have a synergistic therapeutic effect which can overcome the limitations of each single treatment [94]. When cells are subjected to temperatures above 41 °C, not only protein denaturation is occurring but also temporary cell inactivation that could last for several hours [25]. Beyond that time, the surviving cells appear resistant to further exposure to such temperatures due to thermotolerance. For instance, two heating treatments with an interval of 12–48 h can cause an increased blood flow in the tumor, compared with heating with no interval that can decrease the tumor perfusion [95]. Whereas, increased thermotolerance may limit the efficiency of thermal treatment, this could be an advantage for radiation therapy. The appearance of cell thermotolerance, in some cases, can be accompanied by a modification of the cellular response to increase their sensitivity to X-ray irradiation. PTT can also enhance the effects of RT by attenuating the repair of DSBs caused by RT [96,97]. Clinical results have shown that the combination of hyperthermia and RT (ionizing radiation) can increase the rates from 16% to 26% [25]. Cells in hypoxic, low pH areas and cells in S-phase are both relatively radioresistant. The increased blood flow, caused by hyperthermia, can improve tissue oxygenation, which temporarily increases radiosensitivity [98]. Studies have shown that the addition of thermal treatment to the exposure of radiation for 7 days, may extent the tumor growth up to 17 days [95]. 

**Figure 1:**
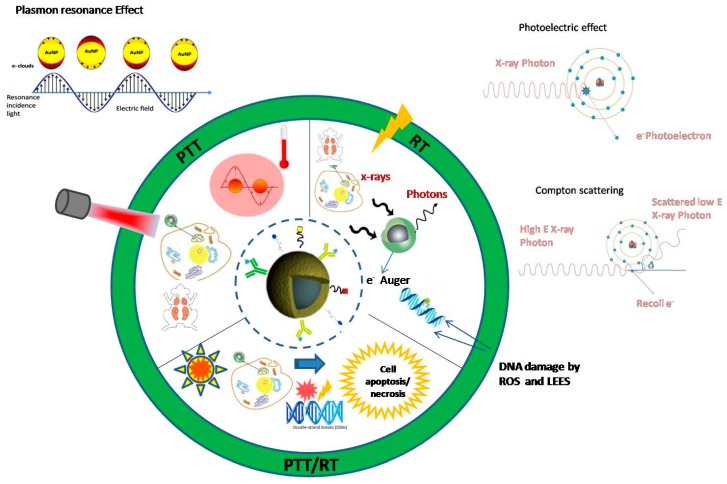
A schematic representation of the basic action mechanisms of photothermal therapy (PTT), ionizing RT and combined PTT/RT in cancer treatment based on functionalized AuNPs.

A basic prerequisite for these highly innovative treatments, before entering clinical applications, is the in-depth knowledge of the biophysical mechanisms involved and the exact dosimetry of the radiation-based treatment method used. In addition, there is accumulating evidence of the strong interaction of AuNPs with the immune system [99] and also that hyperthermia therapy can activate systemic anti-tumor immune responses that can slow growth of untreated tumors [99]. Figure 1 shows a schematic representation of the action mechanisms of PT, RT and PTT/RT in cancer treatment based on functionalized gold NPs.

The co-treatment with PTT and RT based on AuNPs mediated radiosensitization has recently attracted high attention as a very promising dual mode therapy. Zhang and co-workers [100] have demonstrated the synergistic effect of gold nanocages in hyperthermia and RT. They synthesized CD44 Antibody-Conjugated PEG-Modified gold nanocages for 4T1 cells targeting. According to clonogenic survival assays, the combination of CD44-PEG-GNCs, PTT, and RT exhibited a remarkable inhibition rate (96%) compared to the treatment with RT alone (49%). Intratumorally infused small 15 nm gold NPs into radioresistant subcutaneous squamous cell carcinoma (SCCVII) in mice induce a dose decrease when heated by infrared light followed by X-ray treatment. The cells were first irradiated at 1.5 W/cm^2^ by a halogen lamp with a 780 nm high pass filter yielding a peak output at λ = 820 nm and then were irradiated with X-rays generator operating at 100 kVp with a dose rate 7.5 Gy/min at the tumor center. It was found that the dose required to control 50% of the tumors, normally 55 Gy, could be reduced to <15 Gy (a factor of >3.7) [52]. Table 1 provides a list of some indicative publications on different forms of AuNPs that have been used with IR or NIR therapy to treat tumors/cancer cells both in vitro and in vivo. 

As can be seen from the published works, the irradiation parameters coupling with AuNPs properties (such as size, morphology, functionalization and concentration) vary significantly and diverse strategies have been developed for PTT and PTT/RT treatments based on the nanosystems, regarding the laser beams and the ionizing radiation beams of each research group. There is still the requirement for optimized protocols in photothermal treatment and photothermal/radiation synergistic treatments for a translation to the clinical practice. 

## 4. Conclusions

A large number of in vivo and in vitro studies have been conducted based on AuNP-mediated photon- and radiosensitization in PTT and RT. Different approaches and protocols have been applied regarding the size, shape, concentration, and functionalization of AuNPs and the irradiation conditions. The novel combination of PTT with the common RT, based on AuNP dual-mode agents can have a synergistic therapeutic effect which might overcome the limitations of each single treatment, minimizing the curative doses. However, there is still need for optimized protocols in photothermal treatment, radiotherapy and photothermal/radiation synergetic treatment for a translation to clinical practice. The ideal requirements for designing an effective NP as a diagnostic and therapeutic tool are: controlled particle size, surface modification, functionalization, binding behavior, solubility to aqueous environment, low toxicity, biocompatibility, targeting, permeability, retention and biodegradation. The open challenges for effective cancer treatment coupled with the above requirements are still: the immunoactivation and immunosuppression modulation, the tissue thermotolerance, the radioresistance of cancer cells though proteins upregulation and the fully understanding of the biological mechanisms of nanoparticle-mediated radiosensitization. 

## Figures and Tables

**Table 1:** Recent advancements in PTT, RT and PTT/RT and characteristics of testing (cells, AuNP type, concentrations, uptake time, therapy type).

Cellline	AuNP-Type	[CNP]	Up Take Time/Irradiation Time	Therapy Type	Shape	Size (nm)	Reference
*Murine SCK* tested in mice	TNF AuNPs	0.1 mL/20 g bodyweight	1 min	690 nm Pulsed laser 0.1–1 J/cm^2^	spheres	33 nm	[37]
*B16-F10* in vivo tested in nude mice	Doxorubicin-AuNPs	1 μM	24 h/5 min	660 nm laser 8–20 W/cm^2^	sphere	20 nm	[45]
*MIA PaCa-2 and PANC-1* cells	GNS-L/GB	50 μg Au_equiv_	48 h/3 min	808 nm laser 4 W/cm^2^	shells	85 nm	[34]
*MCF-7* tested female BALB/c mice	PEG-AuNSs-Transferrin	1 mg/mL	6 h/3 min	808 nm laser 2 W/cm^2^	shells	30 nm	[46]
*SK-BR-3* tested in mice	PEG-AuPs	7.3 nM	30 min/4–6 min	CW 820 nm 4 W/cm^2^	shells	110 (core) + 10 nm (shell)	[35]
*4T1* cancer cells tested in mice	MPCM-AuNSs	1 mg/mL	20 min/5 min	CW 808 nm 1 W/cm^2^	shells	80(core) + 7 nm (shell)	[36]
*U87MG* tumorstested in nude mice	HGN-siHsp70	1.5 × 10^−9^ M/Kg	90 min/8 min	CW 808 nm 4 W/cm^2^	shells	70 nm	[38]
*SK-BR3*	Anti-HER2-AuNPs	-	5 min	800 nm Pulsed laser 1.6 W/cm^2^	nanocages	65 nm	[47]
*MDA-MB-435* tested in nude mice	PEG-AuNP	14 μg/mL	72 h/5 min	CW 810 nm laser 2 W/cm^2^	nanorods	Aspect ratio 3.7	[39]
*Hs578T, HCC-38, MDA-MB-468 and MDAMB-231*	anti-EGFR-GN	0.22 μg/mL	48 h/3 min	820 nm halogen lamp 1.5 W/cm^2^	nanorods	Aspect ratio 4.0	[43]
*PC3* cells tested in nude mice	Zn(II)/DPA-AuNR	250 μL	24 h/10 min	808 nm 0.5 W/cm^2^	nanorods	Length 51.13 ± 5.2 nm	[44]
*MDA-MB-231*	Thiol-AuNP	12 μM	24 h	X-radiation 160 kVp 4 Gy	sphere	1.9	[48]
*ΕΜΤ-6*	PEG-AuNP	500 μΜ	48 h	X-radiation 10 Gy	sphere	6.1	[46]
*HeLa*	folic Acid-AuNP	255 μΜ	6, 12, 24, 48 h	X-radiation 180 kVp	_	50	[49]
*MCF-7*	Glu-AuNP	100 μM	2 h	X-radiation 100 kVp 10 Gy	sphere	16	[50]
*MDA-MB-361*	anti-HER2-PEG-AuNP	4.8 mg/(g tumor)	48 h	X-radiation 100 kVp 11 Gy	sphere	30	[51]
*SCCVII* tested in mice	PEG-AuNPs	1 μg/mL	24 h/5 min	820 nm halogen lamp 1.5 W/cm^2^ 15 Gy	spheres	15 nm	[52]
*PC-3* cells tested in Foxn1 mice	Goserelin-PEG-AuNRs	0.1–10 μg/g body weight *	72 h/5 min	X-radiation 6 MV 5 Gy	nanorods	_	[53]
*4T1* cells	CD44-Antibody-PEG-AuNPs	3 nM	24 h	CW 808 nm 2.5 W/cm^2^ X-radiation With 2, 4, 6, or 8 Gy of 6-MV	nanocages	58.4 nm	[54]

* The molar concentration μg/g body weight is referred to mg of Au per gram of body weight.
